# Supplementary material for: Size matters: Large copy number losses in Hirschsprung disease patients reveal genes involved in enteric nervous system development
Source: PLoS Genet. 2021 Aug 6;17(8):e1009698. doi: 10.1371/journal.pgen.1009698 (PMC8372947; doi:10.1371/journal.pgen.1009698)
Supplement: S1 Fig — Depicted are the Z-score distributions of all rare CNVs (black lines), rare CN losses (red lines) and CN gains (blue lines). Controls in continuous lines, all HSCR patients in dotted lines. Limited negative z-scores due to the size limit cut-off of 20kb. The z-score distributions of CNV sizes were comparable between groups strongly suggesting that the outliers are responsible for the statistical significant groupwise-differences. (DOCX) [file pgen.1009698.s001.docx]

# S1 Figure: The Z-score distributions of total CNV size, CN loss and CN gain of controls and HSCR patients.
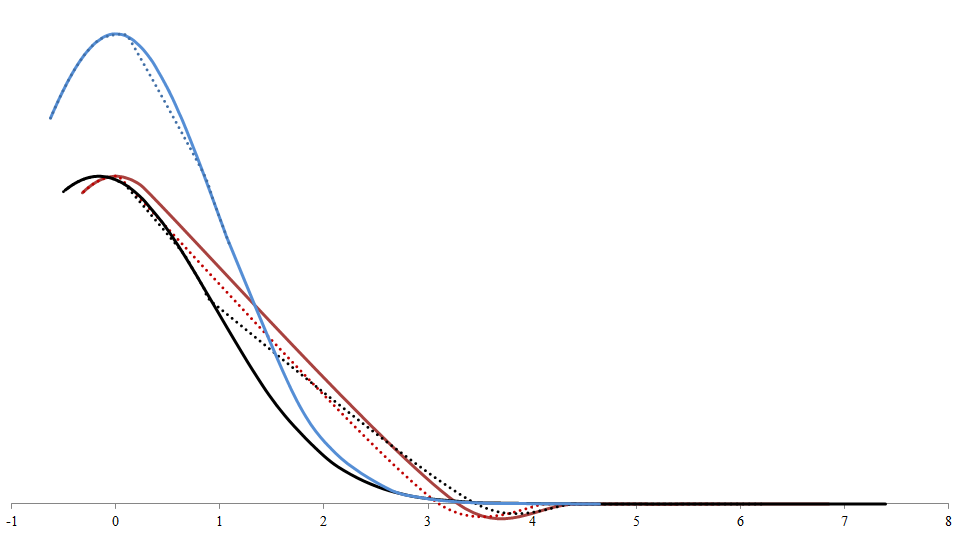
 *Depicted are the Z-score distributions of all rare CNV (black lines), rare CN losses (red lines) and CN gains (blue lines). Controls in continuous lines, all HSCR patients in dotted lines. Limited negative z-scores due to the size limit cut-off of 20kb. The z-score distributions of CNV sizes were comparable between groups strongly suggesting that the outliers are responsible for the statistical significant groupwise-differences.*
