# Supplementary material for: Size matters: Large copy number losses in Hirschsprung disease patients reveal genes involved in enteric nervous system development
Source: PLoS Genet. 2021 Aug 6;17(8):e1009698. doi: 10.1371/journal.pgen.1009698 (PMC8372947; doi:10.1371/journal.pgen.1009698)
Supplement: S2 Fig — Depicted are the calculated noncoding risk scores (RSnc) of patients using the genotypes derived from the 850K array platform. However, we could only do this for the RET and NRG1, as no suitable proxy SNPs were present on the HCS 850k platform for the SEMA3C/D haplotypes (S2 Fig). Therefore, we used (proxy) SNPs present on the GSAMD-v1 chip to determine the Rotterdam population background for all risk haplotypes (group 5, n = 727, Panel B in S2 Fig). (DOCX) [file pgen.1009698.s002.docx]

# S2 Fig: Risk score comparison between Sanger sequenced and SNP-array genotyped samples


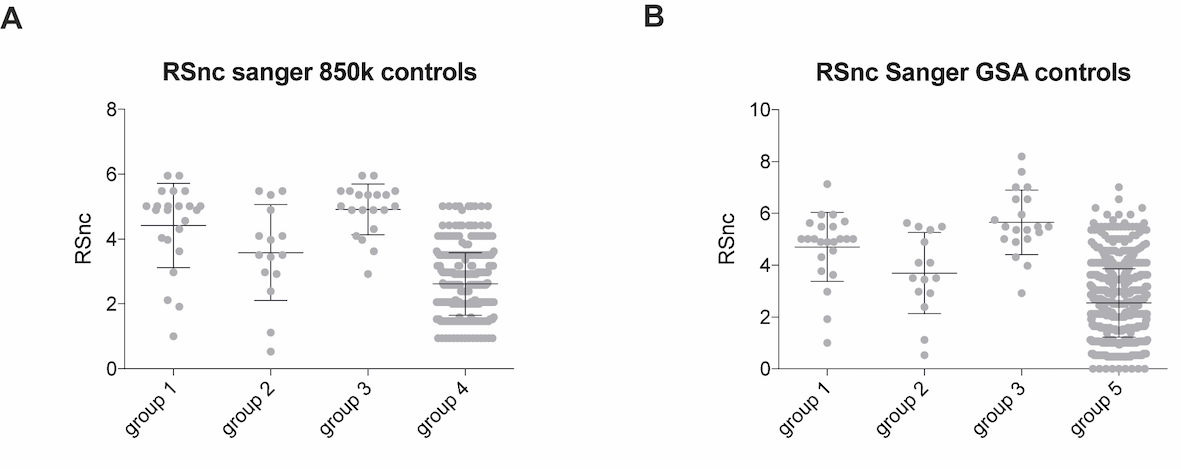


*Depicted are the calculated noncoding risk scores (RSnc) of patients using the genotypes derived from the 850K array platform. However, we could only do this for the RET and NRG1, as no suitable proxy SNPs were present on the HCS 850k platform for the SEMA3C/D haplotypes (S7b). Therefore, we used (proxy) SNPs present on the GSAMD-v1 chip to determine the Rotterdam population background for all risk haplotypes (group 5, n=727, Panel B of S2 Fig).*
