# Supplementary material for: Size matters: Large copy number losses in Hirschsprung disease patients reveal genes involved in enteric nervous system development
Source: PLoS Genet. 2021 Aug 6;17(8):e1009698. doi: 10.1371/journal.pgen.1009698 (PMC8372947; doi:10.1371/journal.pgen.1009698)
Supplement: S3 Fig — Brightfield images of zebrafish larvae injected with gRNAs targeting slc8a1a/b showing severe phenotypes including heart edema, absence of swim bladder and small eyes. Scale bar = 500μm. (DOCX) [file pgen.1009698.s003.docx]

# S3 Fig: Brightfield images of zebrafish larvae injected with *slc8a1* gRNAs (*slc8a1a/slc8a1b*)


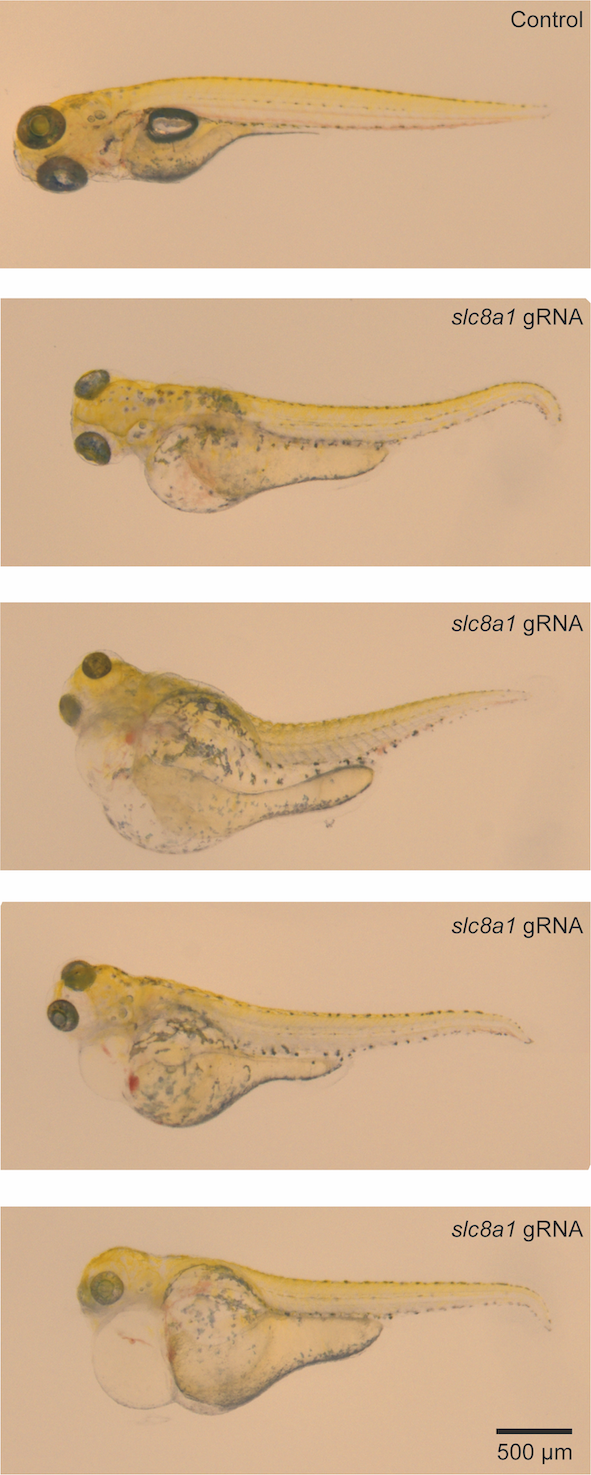


*Brightfield images of zebrafish larvae injected with gRNAs targeting slc8a1a and slc8a1b showing severe phenotypes including heart edema, absence of swim bladder and small eyes. Scale bar = 500µm.*
