# Supplementary material for: Size matters: Large copy number losses in Hirschsprung disease patients reveal genes involved in enteric nervous system development
Source: PLoS Genet. 2021 Aug 6;17(8):e1009698. doi: 10.1371/journal.pgen.1009698 (PMC8372947; doi:10.1371/journal.pgen.1009698)
Supplement: S4 Fig — Brightfield images of zebrafish larvae injected with gRNAs targeting gnl1, tubb5, ufd1l, usp32, akt3a/b, tbx2a/b, gabbr1a/b and mapk8a/b showing mild phenotypes mainly consisting of the absence of a swim bladder and a down bend tail in case of ufd1l disruption. Scale bar = 500μm. (DOCX) [file pgen.1009698.s004.docx]

# S4 Fig: Brightfield images of zebrafish larvae injected with gRNAs

*Brightfield images of zebrafish larvae injected with gRNAs targeting gnl1, tubb5, ufd1l, usp32, akt3a/b, tbx2a/b, gabbr1a/b and mapk8a/b showing mild phenotypes mainly consisting of the absence of a swim bladder and a down bend tail in case of ufd1l disruption. Number of fish are depicted as xx/yy where xx represents the number of fish presenting with the phenotype as depicted in the image and yy represents the total group size. Scale bar = 500µm.*
