# Supplementary material for: Size matters: Large copy number losses in Hirschsprung disease patients reveal genes involved in enteric nervous system development
Source: PLoS Genet. 2021 Aug 6;17(8):e1009698. doi: 10.1371/journal.pgen.1009698 (PMC8372947; doi:10.1371/journal.pgen.1009698)
Supplement: S1 Table — (DOCX) [file pgen.1009698.s005.docx]

**S1 Table: Rare CNVs detected in this cohort**

| ID | Chromosome Region | Length | Cytoband | Probes | Classification | Inheritance | Gender | Group | ClinVar ID |
| --- | --- | --- | --- | --- | --- | --- | --- | --- | --- |
| P_000479 | NC_000001.10:g.3776519_4049451dup | 272933 | p36.32 | 57 | VUS | undetermined | M | 2 | SCV001426225 |
| P_000544 | NC_000001.10:g.25715675_25744764dup | 29090 | p36.11 | 26 | VUS | undetermined | M | 2 | SCV001426226 |
| P_001636 | NC_000001.10:g.25715675_25744764dup | 29090 | p36.11 | 26 | VUS | undetermined | M | 3 | SCV001426226 |
| P_000566 | NC_000001.10:g.25717366_25744764dup | 27399 | p36.11 | 24 | VUS | undetermined | M | 2 | SCV001426227 |
| P_000498 | NC_000001.10:g.152286216_152323703dup | 37488 | q21.3 | 11 | VUS | undetermined | F | 3 | SCV001426228 |
| P_000450 | NC_000001.10:g.185109784_185132629dup | 22846 | q25.3 | 41 | VUS | undetermined | M | 3 | SCV001426229 |
| P_002431 | NC_000001.10:g.243963527_244016804del | 53278 | q44 | 9 | likely deleterious | undetermined | F | 3 | SCV001426230 |
| P_001637 | NC_000002.11:g.10664398_10914786dup | 250389 | p25.1 | 70 | VUS | undetermined | M | 3 | SCV001426231 |
| P_000479 | NC_000002.11:g.40624267_40646501del | 22235 | p22.1 | 11 | likely deleterious | undetermined | M | 2 | SCV001426232 |
| P_000582 | NC_000002.11:g.102658576_102847088dup | 188513 | q11.2 - q12.1 | 57 | VUS | undetermined | M | 3 | SCV001426233 |
| P_000567 | NC_000002.11:g.177128475_177259979dup | 131505 | q31.1 | 15 | VUS | undetermined | M | 1 | SCV001426234 |
| P_000557 | NC_000002.11:g.189848997_189872988del | 23992 | q32.2 | 53 | VUS | undetermined | M | 2 | SCV001426235 |
| P_000557 | NC_000002.11:g.206875802_207000559del | 124758 | q33.3 | 18 | VUS | undetermined | M | 2 | SCV001426236 |
| P_000573 | NC_000002.11:g.216214577_216299733del | 85157 | q35 | 49 | VUS | undetermined | F | 1 | SCV001426237 |
| P_000490 | NC_000002.11:g.220283220_220284269del | 1050 | q35 | 9 | VUS | undetermined | F | 3 | SCV001426238 |
| P_000302 | NC_000003.11:g.14406477_14509088dup | 102612 | p25.1 | 53 | likely deleterious | undetermined | F | 2 | SCV001426239 |
| P_000515 | NC_000003.11:g.57414966_57492375del | 77410 | p14.3 | 15 | VUS | undetermined | M | 3 | SCV001426240 |
| P_000579 | NC_000003.11:g.60468409_60490104del | 21696 | p14.2 | 16 | likely deleterious | undetermined | M | 3 | SCV001426241 |
| P_000512 | NC_000003.11:g.108481092_108926543del | 445452 | q13.13 | 30 | VUS | maternal | F | 1 | SCV001426242 |
| P_000480 | NC_000003.11:g.137727844_137778355del | 50512 | q22.3 | 14 | VUS | undetermined | M | 2 | SCV001426243 |
| P_002431 | NC_000003.11:g.145774557_145801149dup | 26593 | q24 | 9 | VUS | undetermined | F | 3 | SCV001426244 |
| P_000557 | NC_000004.11:g.159541187_159631380del | 90194 | q32.1 | 48 | VUS | undetermined | M | 2 | SCV001426245 |
| P_000515 | NC_000004.11:g.159596032_159621356del | 25325 | q32.1 | 20 | VUS | undetermined | M | 3 | SCV001426246 |
| P_001639 | NC_000006.11:g.22008230_22093109del | 84880 | p22.3 | 32 | VUS | undetermined | M | 3 | SCV001426247 |
| P_000512 | NC_000006.11:g.28005012_31683185del | 3678174 | p22.1 - p21.33 | 403 | likely deleterious | de novo | F | 1 | SCV001426248 |
| P_000490 | NC_000006.11:g.49662135_49664605del | 2471 | p12.3 | 6 | VUS | undetermined | F | 3 | SCV001426249 |
| P_002455 | NC_000007.13:g.3627221_3759274del | 132054 | p22.2 | 32 | VUS | undetermined | M | 1 | SCV001426250 |
| P_000582 | NC_000007.13:g.4736454_4860123dup | 123671 | p22.1 | 47 | VUS | undetermined | M | 3 | SCV001426251 |
| P_000582 | NC_000007.13:g.4929022_5218030dup | 289009 | p22.1 | 59 | VUS | undetermined | M | 3 | SCV001426252 |
| P_000582 | NC_000007.13:g.5239584_5401976dup | 162393 | p22.1 | 54 | VUS | undetermined | M | 3 | SCV001426253 |
| P_002431 | NC_000007.13:g.95845896_96004178del | 158283 | q21.3 | 16 | VUS | undetermined | F | 3 | SCV001426254 |
| P_000490 | NC_000007.13:g.117233848_117237342del | 3495 | q31.2 | 13 | VUS | undetermined | F | 3 | SCV001426255 |
| P_000490 | NC_000007.13:g.117287774_117293718del | 5945 | q31.2 | 6 | VUS | undetermined | F | 3 | SCV001426256 |
| P_000555 | NC_000007.13:g.151797921_152258693dup | 460773 | q36.1 | 52 | VUS | de novo | F | 1 | SCV001426257 |

**S1 Table: Rare CNV detected in this cohort (continued)**

| ID | Chromosome Region | Length | Cytoband | Probes | Classification | Inheritance | Gender | Group | ClinVar ID |
| --- | --- | --- | --- | --- | --- | --- | --- | --- | --- |
| P_000568 | NC_000008.10:g.95186036_95301703dup | 115668 | q22.1 | 41 | VUS | undetermined | M | 1 | SCV001426258 |
| P_002450 | NC_000009.11:g.28393380_28462962del | 69583 | p21.1 | 21 | VUS | undetermined | M | 1 | SCV001426259 |
| P_000537 | NC_000010.10:g.49033586_52417694del | 3384109 | q11.22 - q11.23 | 183 | likely deleterious | maternal | M | 1 | SCV001426260 |
| P_000557 | NC_000011.9:g.62251301_62298871dup | 47571 | q12.3 | 25 | likely deleterious | undetermined | M | 2 | SCV001426261 |
| P_000479 | NC_000012.11:g.9245492_9308543dup | 63052 | p13.31 | 24 | VUS | undetermined | M | 2 | SCV001426262 |
| P_000561 | NC_000012.11:g.80598919_80836812del | 237894 | q21.31 | 12 | VUS | undetermined | M | 1 | SCV001426263 |
| P_002459 | NC_000012.11:g.94767704_94880489del | 112786 | q22 | 27 | VUS | undetermined | M | 1 | SCV001426264 |
| P_000494 | NC_000012.11:g.128208742_128917555dup | 708814 | q24.32 | 96 | VUS | paternal | M | 1 | SCV001426265 |
| P_000544 | NC_000015.9:g.62409198_62521004dup | 111807 | q22.2 | 33 | VUS | undetermined | M | 2 | SCV001426266 |
| P_000502 | NC_000015.9:g.80527215_80603142dup | 75928 | q25.1 | 22 | VUS | undetermined | F | 2 | SCV001426267 |
| P_000567 | NC_000017.10:g.58076721_60362868del | 2286148 | q23.1 - q23.2 | 74 | likely deleterious | de novo | M | 1 | SCV001426268 |
| P_000552 | NC_000018.9:g.6335542_6430944dup | 95403 | p11.31 | 20 | VUS | undetermined | M | 3 | SCV001426269 |
| P_000552 | NC_000018.9:g.6454093_6548624dup | 94532 | p11.31 | 37 | VUS | undetermined | M | 3 | SCV001426270 |
| P_000520 | NC_000018.9:g.45755986_45787673dup | 31688 | q21.1 | 13 | VUS | undetermined | M | 1 | SCV001426271 |
| P_001763 | NC_000018.9:g.65699090_66534856dup | 835767 | q22.1 | 228 | VUS | undetermined | F | 1 | SCV001426272 |
| P_000514 | NC_000021.8:g.47819478_47844620del | 25143 | q22.3 | 14 | VUS | undetermined | M | 3 | SCV001426273 |
| P_001632 | NC_000022.10:g.18687210_19060954dup | 373745 | q11.21 | 75 | VUS; modifier | undetermined | M | 3 | SCV001426274 |
| P_000561 | NC_000022.10:g.18861209_21630630del | 2769422 | q11.21 | 446 | likely deleterious | de novo | M | 1 | SCV001426275 |
| P_002455 | NC_000022.10:g.21802791_22555544dup | 752756 | q11.21 - q11.22 | 169 | VUS | de novo | M | 1 | SCV001426276 |
| P_000573 | NC_000023.10:g.3184901_3240953dup | 56053 | p22.33 | 16 | VUS | undetermined | F | 1 | SCV001426277 |
| P_000579 | NC_000023.10:g.94409037_94446394del | 37358 | q21.33 | 5 | VUS | undetermined | M | 3 | SCV001426278 |
| P_000540 | NC_000023.10:g.154277428_154299482del | 22055 | q28 | 5 | VUS | maternal | M | 1 | SCV001426279 |
