# Supplementary material for: Size matters: Large copy number losses in Hirschsprung disease patients reveal genes involved in enteric nervous system development
Source: PLoS Genet. 2021 Aug 6;17(8):e1009698. doi: 10.1371/journal.pgen.1009698 (PMC8372947; doi:10.1371/journal.pgen.1009698)
Supplement: S2 Table — The number and size of rare CNVs, the number of rare losses and gains, the number of genes considered a CCR, the number of ENS genes per rare CNV, and the relative weighted risk score, were determined and compared for the different groups with a single ANOVA test. If group differences existed (p<0.05), we determined which subgroups were significantly different, using a two-tailed T-test. Abbreviations: CCR; Constrained Coding Region; SNV; single nucleotide variant, CNV; Copy Number Variation, ENS; Enteric Nervous System, RSnc; Risk Score non coding, ND; not determined. Higher values in green, lower values in red. Two-tailed p-values. (DOCX) [file pgen.1009698.s006.docx]

**S2 Table: Statistical comparisons**

|  | **F** | **P** | **Fcrit** | **group 1 vs group 2** | **group 1 vs group 3** | **group 2 vs group 3** | **Group 1 vs group 4** | **Group 2 vs group 4** | **Group 3 vs group 4** | | **group 1 (n=23)** | **group 2 (n=15)** | **group 3 (n=20)** | **group 4 (n=326)** | **group 5 (n=727)** |
| --- | --- | --- | --- | --- | --- | --- | --- | --- | --- | --- | --- | --- | --- | --- | --- |
| **rare CNV Size** | 8,519 | 1,73E-05 | 2,628 | 5,58E-02 | 3,91E-02 | 4,68E-01 | 3,25E-06 | 4,62E-01 | 6,47E-01 |  | 708952 | 69472 | 109500 | 156518 | ND |
| **rare Loss Size** | 10,043 | 2,21E-06 | 2,628 | 1,00E-01 | 5,90E-02 | 8,38E-01 | 3,64E-07 | 7,05E-01 | 7,00E-01 |  | 574906 | 20779 | 24817 | 55062 | ND |
| **ENS genes in a rare CNV** | 11,372 | 3,70E-07 | 2,628 | 8,75E-02 | 7,02E-02 | 5,26E-01 | 4,93E-08 | 5,49E-01 | 9,40E-01 |  | 3,22 | 0,33 | 0,55 | 0,58 | ND |
| **ENS genes in a rare Loss** | 15,483 | 1,60E-09 | 2,628 | 1,08E-01 | 7,16E-02 | 5,54E-01 | 3,26E-10 | 5,45E-01 | 7,61E-01 |  | 2,78 | 0,07 | 0,15 | 0,21 | ND |
| **CCR in a rare CNV** | 6,415 | 3,01E-04 | 2,628 | 2,75E-01 | 1,38E-01 | 2,45E-01 | 4,63E-05 | 9,66E-01 | 3,45E-01 |  | 2,35 | 0,47 | 0,15 | 0,45 | ND |
| **CCR in a rare loss** | 7,566 | 6,29E-05 | 2,628 | 2,80E-01 | 1,62E-01 | 2,55E-01 | 8,13E-06 | 5,78E-01 | 5,78E-01 |  | 2,13 | 0,27 | 0,05 | 0,19 | ND |
| **ENS and CCR in a rare CNV** | 3,202 | 2,33E-02 | 2,628 | 5,20E-01 | 1,79E-01 | 3,50E-01 | 3,40E-03 | 3,37E-01 | 5,67E-01 |  | 0,48 | 0,27 | 0,10 | 0,16 | ND |
| **ENS and CCR in a rare Loss** | 4,720 | 3,01E-03 | 2,628 | 2,78E-01 | 2,82E-01 | 7,98E-01 | 2,20E-04 | 9,77E-01 | 6,21E-01 |  | 0,39 | 0,07 | 0,10 | 0,06 | ND |
| **RSnc with Sanger sequenced cases and with HCS850k genotyped controls** | 55,511 | 8,89E-30 | 2,628 | 7,53E-02 | 1,43E-01 | 1,53E-03 | 8,99E-16 | 2,56E-04 | 2,29E-22 |  | 4,42 | 3,59 | 4,92 | 2,62 | ND |
| **RSnc with Sanger sequenced cases and with GSA genotyped controls** | 56,570 | 4,26E-33 | 2,616 | 4,03E-02 | 2,08E-02 | 2,29E-04 | 4,60E-14 | 9,61E-04 | 1,22E-23 |  | 4,71 | 3,70 | 5,66 | ND | 2,54 |
| **RSnc with GSA genotyped cases and controls** | 43,649 | 4,79E-26 | 2,617 | 1,37E-01 | 3,54E-01 | 1,27E-02 | 4,06E-08 | 4,94E-05 | 9,50E-20 |  | 4,88 | 4,06 | 5,36 | ND | 2,54 |

*The number and size of rare CNVs, the number of rare losses and gains,* *the number of genes considered a CCR, the number of ENS genes per rare CNV, and the relative weighted risk score, were determined and compared for the different groups with a single ANOVA test. If group differences existed (p<0.05), we determined which subgroups were significantly different, using a two-tailed T-test. Abbreviations: CCR; Constrained Coding Region, SNV; single nucleotide variant, CNV; Copy Number Variation, ENS; Enteric Nervous System, RSnc; Risk Score non coding, ND; not determined. Higher values in green, lower values in red. Two-tailed p-values.*
