# Supplementary material for: Size matters: Large copy number losses in Hirschsprung disease patients reveal genes involved in enteric nervous system development
Source: PLoS Genet. 2021 Aug 6;17(8):e1009698. doi: 10.1371/journal.pgen.1009698 (PMC8372947; doi:10.1371/journal.pgen.1009698)
Supplement: S3 Table — Most known HSCR genes are intolerant to genetic variation and are rarely impacted by CNVs in unaffected individuals [39, 40]. These genes have been described to be impacted by CNVs in HSCR patients [7, 27], although this does not seem to be a frequent phenomenon, as in our cohort we did not detect any CNV impacting a known HSCR gene. Abbreviations: mis_z: Missense variation Z-score, syn_z: Synonymous variation z-sore, PLI: probability of being loss-of-function intolerant, PRec: probability of being tolerant to heterozygous loss of function variation but intolerant to homozygous loss of function variation, PNull: probability of being tolerant to loss of function variation (heterozygous or homozygous), del: deletion, dup: duplication, EW: embryonic week, DDD: deciphering developmental disorders project. Data derived from (https://gnomad.broadinstitute.org/) and the DDD control track at: https://genome-euro.ucsc.edu/. (DOCX) [file pgen.1009698.s007.docx]

**S3 Table: HSCR disease gene characteristics**

*Most known HSCR genes are intolerant to genetic variation and are rarely impacted by CNVs in unaffected individuals[1, 2]. These genes have been described to be impacted by CNVs in HSCR patients[3, 4], although this does not seem to be a frequent phenomenon, as in our cohort we did not detect any CNV impacting a known HSCR gene. Abbreviations: mis_z: Missense variation Z-score, syn_z:*  *Synonymous variation z-sore****,*** *PLI****:*** *probability of being loss-of-function intolerant , PRec: probability of being tolerant to heterozygous loss of function variation but intolerant to homozygous loss of function variation, PNull: probability of being tolerant to loss of function variation (heterozygous or homozygous), del: deletion, dup: duplication, EW: embryonic week, DDD: deciphering developmental disorders project. Data derived from (https://gnomad.broadinstitute.org/) and the DDD control track at: https://genome-euro.ucsc.edu/*

**References**

1. Coe BP, Witherspoon K, Rosenfeld JA, van Bon BW, Vulto-van Silfhout AT, Bosco P, et al. Refining analyses of copy number variation identifies specific genes associated with developmental delay. Nature genetics. 2014;46(10):1063-71. PubMed PMID: 25217958.

2. Cooper GM, Coe BP, Girirajan S, Rosenfeld JA, Vu TH, Baker C, et al. A copy number variation morbidity map of developmental delay. Nature genetics. 2011;43(9):838-46. PubMed PMID: 21841781.

3. Amiel J, Espinosa-Parrilla Y, Steffann J, Gosset P, Pelet A, Prieur M, et al. Large-scale deletions and SMADIP1 truncating mutations in syndromic Hirschsprung disease with involvement of midline structures. Am J Hum Genet. 2001;69(6):1370-7. PubMed PMID: 11595972.

4. Amiel J, Sproat-Emison E, Garcia-Barcelo M, Lantieri F, Burzynski G, Borrego S, et al. Hirschsprung disease, associated syndromes and genetics: a review. J Med Genet. 2008;45(1):1-14. PubMed PMID: 17965226.
