# Supplementary material for: Size matters: Large copy number losses in Hirschsprung disease patients reveal genes involved in enteric nervous system development
Source: PLoS Genet. 2021 Aug 6;17(8):e1009698. doi: 10.1371/journal.pgen.1009698 (PMC8372947; doi:10.1371/journal.pgen.1009698)
Supplement: S4 Table — (DOCX) [file pgen.1009698.s008.docx]

**S4 Table: Rare CNV with “ENS genes”**

| **P-number** | **Chromosome Region** | **Event** | **Length** | **Cytoband** | **Probes** | **Class** | **Sex** | **ENS gene(s)** |
| --- | --- | --- | --- | --- | --- | --- | --- | --- |
| **P_000302** | chr3:14,406,477-14,509,088 | CN Gain | 102612 | p25.1 | 53 | LD | F | ***SLC6A6*** |
| **P_000479** | chr12:9,245,492-9,308,543 | CN Gain | 63052 | p13.31 | 24 | VUS | M | ***A2M*** |
| **P_000479** | chr2:40,624,267-40,646,501 | CN Loss | 22235 | p22.1 | 11 | LD | M | ***SLC8A1*** |
| **P_000494** | chr12:128,208,742-128,917,555 | CN Gain | 708814 | q24.32 | 96 | VUS | M | *TMEM132C* |
| **P_000498** | chr1:152,286,216-152,323,703 | CN Gain | 37488 | q21.3 | 11 | VUS | F | *FLG* |
| **P_000502** | chr15:80,527,215-80,603,142 | CN Gain | 75928 | q25.1 | 22 | VUS | F | *CTXND1* |
| **P_000512** | chr6:28,005,012-31,683,185 | CN Loss | 3678174 | p22.1 - p21.33 | 403 | LD | F | *6M1-18, ABHD16A, ATAT1, ATP6V1G2, DDR1,* ***DPCR1****, FLOT1,* ***GABBR1****,* ***GNL1****, HLA-H, IER3, MUCL3, OR11A1, OR2J2, OR2J3, PGBD1, PPP1R11, PP1R18,* ***TUBB****, ZKSCAN4, ZNRD1-AS1, ZNRD1ASP,* ***ZSCAN31*** |
| **P_000520** | chr18:45,755,986-45,787,673 | CN Gain | 31688 | q21.1 | 13 | VUS | M | *ZBTB7C* |
| **P_000537** | chr10:49,033,586-52,417,694 | CN Loss | 3384109 | q11.22 - q11.23 | 183 | LD | M | *ARHGAP22, C10orf128, CHAT, FAM21A,* ***MAPK8****, NCOA4, SLC18A3, TIMM23, TIMM23B, TMEM273, VSTM4, WASHC2A* |
| **P_000540** | chrX:154,277,428-154,299,482 | Hemizygous | 22055 | q28 | 5 | VUS | M | *CMC4, FUNDC2, MTCP2* |
| **P_000557** | chr11:62,251,301-62,298,871 | CN Gain | 47571 | q12.3 | 25 | LD | M | ***AHNAK*** |
| **P_000561** | chr22:18,861,209-21,630,630 | CN Loss | 2769422 | q11.21 | 446 | LD | M | *ARVCF, BCRP2, C22orf29, C22orf39, CDC45, COMT, DGCR14, DGCR2, ESS2, GP1BB, LZTR1, RIMBP3, RTL10, SLC7A4, UFD1,* ***UFD1L****, ZDHHC8, ZNF74* |
| **P_000567** | chr17:58,076,721-60,362,868 | CN Loss | 2286148 | q23.1 - q23.2 | 74 | LD | M | *BCAS3, HEATR6,* ***TBX2****,* ***USP32*** |
| **P_000573** | chr2:216,214,577-216,299,733 | CN Loss | 85157 | q35 | 49 | VUS | F | *FN1* |
| **P_000579** | chr3:60,468,409-60,490,104 | CN Loss | 21696 | p14.2 | 16 | LD | M | *FHIT* |
| **P_000582** | chr7:4,929,022-5,218,030 | CN Gain | 289009 | p22.1 | 59 | VUS | M | *MMD2* |
| **P_000582** | chr2:102,658,576-102,847,088 | CN Gain | 188513 | q11.2 - q12.1 | 57 | VUS | M | *IL1R1, IL1RL2* |
| **P_000582** | chr7:5,239,584-5,401,976 | CN Gain | 162393 | p22.1 | 54 | VUS | M | *SLC29A4, WIPI2* |
| **P_001632** | chr22:18,687,210-19,060,954 | CN Gain | 373745 | q11.21 | 75 | VUS | M | *DGCR2; known modifier CNV* |
| **P_001637** | chr2:10,664,398-10,914,786 | CN Gain | 250389 | p25.1 | 70 | VUS | M | *NOL10* |
| **P_001763** | chr18:65,699,090-66,534,856 | CN Gain | 835767 | q22.1 | 228 | VUS | F | *TMX3* |
| **P_002431** | chr1:243,963,527-244,016,804 | CN Loss | 53278 | q44 | 9 | LD | F | ***AKT3*** |
| **P_002431** | chr7:95,845,896-96,004,178 | CN Loss | 158283 | q21.3 | 16 | VUS | F | *SLC25A13* |
| **P_002450** | chr9:28,393,380-28,462,962 | CN Loss | 69583 | p21.1 | 21 | VUS | M | *LINGO2* |
| **P_002455** | chr22:21,802,791-22,555,544 | CN Gain | 752756 | q11.21 - q11.22 | 169 | VUS | M | *CCDC116,* ***MAPK1****, PPM1F, SDF2L1, TMEM191C,* ***YDJC****, YPEL1* |
| **P_002459** | chr12:94,767,704-94,880,489 | CN Loss | 112786 | q22 | 27 | VUS | M | *CCDC41, CEP83* |
| Abbreviations: CN; Copy Number, LD; Likely deleterious, VUS; variant of unknown significance, M; Male, F, Female. In bold “ENS genes” that are a CCR. Chromosomal regions according to build hg19. | | | | | | | | |
