# Supplementary material for: Size matters: Large copy number losses in Hirschsprung disease patients reveal genes involved in enteric nervous system development
Source: PLoS Genet. 2021 Aug 6;17(8):e1009698. doi: 10.1371/journal.pgen.1009698 (PMC8372947; doi:10.1371/journal.pgen.1009698)
Supplement: S5 Table — Abbreviations: NVAR: number of variants, NCASEHET: number of heterozygous variants in cases, NCTRLHET: number of heterozygous variants in controls. (DOCX) [file pgen.1009698.s009.docx]

**S5 Table: Variant prioritization and WGS variant burden test results in genes impacted by rare CNV**

To determine whether a gene affected by a rare putative deleterious CNV was predicted to be a constraint coding region (CCR) [1, 2] we used a threshold of probability of Loss of function Intolerant (pLI > 0.85), synonymous or missense z-score of at least 3 for missense variants. Deletions or duplications: del score ≥ 1, dup score ≥ 1 and cnv score ≥ 1. Additionally, genes impacted more than once by a deletion or a duplication were not considered a CCR. We used capture-specific controls to eliminate technical noise in (1) a WES cohort of sporadic HSCR (n=76, 149 controls) and (2) a Whole Genome Sequencing (WGS) cohort of 443 short segment HSCR patients and 493 unaffected controls [3]. Variants from WES data previously generated, were prioritized as follows: an allele frequency below 1% in *in-house* unaffected controls (n=906); affect a CCR and have an allele frequency of maximum 0.01 for homozygous recessive variants and of 0.001 for heterozygous variants, in GnomAD. We used a CADD score of 20 as a measure for deleteriousness for missense variants. All variants within two bases of an intron-exon boundary were considered to affect splicing, and were included in the “loss of function” category when considering gene constraint. Using RVTESTS[4], a variant burden test was done comparing the variant burden in 443 short segment HSCR patients and 493 controls (WGS)[3] using an allele frequency of maximum 0.01 for homozygous recessive variants and of 0.001 for heterozygous variants, in GnomAD. All rare putative deleterious loss of function variants unique to the HSCR cohort in constraint genes are described in S1 Table and were uploaded to the ClinVar database (<https://www.ncbi.nlm.nih.gov/clinvar/>). Given the sample size and possible variant positions tested we ranked the genes based on p-value and considered a p-value below 1x10e-5 of interest”

*Abbreviations: NVAR: number of variants, NCASEHET: number of heterozygous variants in cases, NCTRLHET: number of heterozygous variants in controls*

**References**

1. Lek M, Karczewski KJ, Minikel EV, Samocha KE, Banks E, Fennell T, et al. Analysis of protein-coding genetic variation in 60,706 humans. Nature. 2016;536(7616):285-91. PubMed PMID: 27535533.

2. Ruderfer DM, Hamamsy T, Lek M, Karczewski KJ, Kavanagh D, Samocha KE, et al. Patterns of genic intolerance of rare copy number variation in 59,898 human exomes. Nature genetics. 2016;48(10):1107-11. Epub 2016/08/18. doi: 10.1038/ng.3638. PubMed PMID: 27533299; PubMed Central PMCID: PMCPMC5042837.

3. Tang CS, Li P, Lai FP, Fu AX, Lau ST, So MT, et al. Identification of Genes Associated With Hirschsprung Disease, Based on Whole-Genome Sequence Analysis, and Potential Effects on Enteric Nervous System Development. Gastroenterology. 2018;155(6):1908-22.e5. Epub 2018/09/16. doi: 10.1053/j.gastro.2018.09.012. PubMed PMID: 30217742.

4. Zhan X, Hu Y, Li B, Abecasis GR, Liu DJ. RVTESTS: an efficient and comprehensive tool for rare variant association analysis using sequence data. Bioinformatics (Oxford, England). 2016;32(9):1423-6. PubMed PMID: 27153000.
