# Supplementary material for: Size matters: Large copy number losses in Hirschsprung disease patients reveal genes involved in enteric nervous system development
Source: PLoS Genet. 2021 Aug 6;17(8):e1009698. doi: 10.1371/journal.pgen.1009698 (PMC8372947; doi:10.1371/journal.pgen.1009698)
Supplement: S6 Table — Highlighted SNPS ingrey/ boldare the risk alleles and Odds ratio’s used in the polygenic risk score calculation. #We did not account for the increased risk of having the two main risk haplotype combinations [1]. @ this risk haplotype was not present in this patient cohort. Sanger sequencing was used to genotype all patients for SNPs known to be associated to HSCR [9, 10, 12, 13]. Patient and controls genotypes were determined using SNPs from the GSAMD-v1 platform. D’ and R2 derived from (https://ldlink.nci.nih.gov/) in European population. Primer sequences can be found in S8 Table. (DOCX) [file pgen.1009698.s010.docx]

**S6 Table: Risk haplotype markers and odds ratio’s used to calculate noncoding risk scores (RSnc)**

| Gene | risk/non-risk allele | Risk allele frequency | Odds ratio (95% CI) | P | OR | GSAMD v1 | risk/non-risk | D' | R^2^ | Ref |
| --- | --- | --- | --- | --- | --- | --- | --- | --- | --- | --- |
| *SEMA3C/D* | **rs11766001:C/A** | **0.22/0.15** | **1.6 (1.3–2.0)** | **1.0 × 10−4** | **1.6** | **rs4374933** | **T/C** | **0.9834** | **0.6221** | **[1]** |
| *SEMA3C/D* | **rs80227144: C/A** | **0.14/0.03** | **5.2 (3.09–8.73)** | **4.0×10-4** | **5.2** | **rs80227144** | **C/A** | **1** | **1** | **[1]** |
| *RET^#^* | **rs2506030: G/A** | **0.56/0.41** | **1.8 (1.5-2.2)** | **4.46×10-11** | **1.8** | **GSA-rs2506030** | **G/A** | **1** | **1** | **[2]** |
| *RET^#^* | **rs7069590: T/C** | **0.84/0.76** | **1.7 (1.4-2.2)** | **4.36×10-6** | **1.7** | **rs71505660** | **C/A** | **0.9946** | **0.984** | **[2]** |
| *RET^#^* | **rs2435357: T/C** | **0.58/0.25** | **4.01 (3.33–4.84)** | **2.98×10-48** | **4.01** | **rs2435344** | **T/C** | **0.9841** | **0.9338** | **[2]** |
| *RET*^@^ | rs9282834: A/G | 0.05/0.03 | 1.80 (1.06–3.04) | 0.029 | 1.8 | NA (to rare) | NA | NA | NA | [3] |
| *RET* | rs2505998: A/C | 0.64/0.22 | 4.17 (3.23–5.26) | 1.1×10-28 | 4.17 | rs2435344 | C/A | 0.9838 | 0.9088 | [3] |
| *NRG1* | **rs7005606: T/G** | **0.54/0.42** | **1.64 (1.25–2.15)** | **4.7×10-10** | **1.64** | **rs4733130** | **T/C** | **1** | **1** | **[3]** |
|  |  |  |  |  |  |  |  |  |  |  |
| Gene | **Risk haplotype** | **Risk haplotype frequency** | **Odds ratio (95% CI)** | **P** | **OR** |  |  |  |  | **Ref** |
| *RET* | ATT# | 0.14/0.08 | 3.13 (2.17-4.50) | 8.31 × 10−10 | - |  |  |  |  | [2] |
| *RET* | GTT# | 0.42/0.16 | 4.40 (3.26-5.94) | 3.62 × 10−22 | - |  |  |  |  | [2] |
| *RET* | TA^@^ | 0.05/0.03 | 20.3 (9.31–44.4) | 2.98×10-48 | - |  |  |  |  | [3] |

*Highlighted SNPS* ***in grey/ bold*** *are the risk alleles and Odds ratio’s used in the polygenic risk score calculation. ^#^We did not account for the increased risk of having the two main risk haplotype combinations [1]. ^@^ this risk haplotype was not present in this patient cohort. Sanger sequencing was used to genotype all patients for SNPs known to be associated to HSCR [1, 2, 4, 5]., Patient and controls genotypes were determined using SNPs from the GSAMD-v1 platform. D’ and R2 derived from (*[*https://ldlink.nci.nih.gov/*](https://ldlink.nci.nih.gov/)*) in European population. Primer sequences can be found in S8 Table.*

**References**

1. Kapoor A, Jiang Q, Chatterjee S, Chakraborty P, Sosa MX, Berrios C, et al. Population variation in total genetic risk of Hirschsprung disease from common RET, SEMA3 and NRG1 susceptibility polymorphisms. Human molecular genetics. 2015;24(10):2997-3003. Epub 2015/02/11. doi: 10.1093/hmg/ddv051. PubMed PMID: 25666438; PubMed Central PMCID: PMCPMC4406299.

2. Chatterjee S, Kapoor A, Akiyama JA, Auer DR, Lee D, Gabriel S, et al. Enhancer Variants Synergistically Drive Dysfunction of a Gene Regulatory Network In Hirschsprung Disease. Cell. 2016;167(2):355-68.e10. Epub 2016/10/04. doi: 10.1016/j.cell.2016.09.005. PubMed PMID: 27693352; PubMed Central PMCID: PMCPMC5113733.

3. Tang CS, Gui H, Kapoor A, Kim JH, Luzon-Toro B, Pelet A, et al. Trans-ethnic meta-analysis of genome-wide association studies for Hirschsprung disease. Human molecular genetics. 2016;25(23):5265-75. Epub 2016/10/06. doi: 10.1093/hmg/ddw333. PubMed PMID: 27702942.

4. Jiang Q, Ho YY, Hao L, Nichols Berrios C, Chakravarti A. Copy number variants in candidate genes are genetic modifiers of Hirschsprung disease. PloS one. 2011;6(6):e21219. Epub 2011/06/30. doi: 10.1371/journal.pone.0021219. PubMed PMID: 21712996; PubMed Central PMCID: PMCPMC3119685.

5. Jiang Q, Arnold S, Heanue T, Kilambi KP, Doan B, Kapoor A, et al. Functional loss of semaphorin 3C and/or semaphorin 3D and their epistatic interaction with ret are critical to Hirschsprung disease liability. Am J Hum Genet. 2015;96(4):581-96. Epub 2015/04/04. doi: S0002-9297(15)00067-1 [pii]

10.1016/j.ajhg.2015.02.014. PubMed PMID: 25839327; PubMed Central PMCID: PMC4385176.
