# Supplementary material for: Size matters: Large copy number losses in Hirschsprung disease patients reveal genes involved in enteric nervous system development
Source: PLoS Genet. 2021 Aug 6;17(8):e1009698. doi: 10.1371/journal.pgen.1009698 (PMC8372947; doi:10.1371/journal.pgen.1009698)
Supplement: S7 Table — Depicted are the individual patient’s characteristics, number of predisposing haplotypes (as represented by the risk SNPs) and the noncoding risk score (RSnc). Additionally, the RET risk haplotypes as described inS6 Tableare depicted. In blue the number of “ENS genes” in a CN gain, in red the number of “ENS genes” in a CN loss. Depicted in blue: (1) functional evidence from zebrafish studies and (2) the number of additional patients containing putative deleterious variants in genes within the rare CNVs. $No phasing was performed to discern the most likely haplotype. (DOCX) [file pgen.1009698.s011.docx]

**S7 Table: Description of individual patient genetic risk profiles**

| **patient** | **segment** | **Sex** | **group** | **rs2506030** | **rs7069590** | **rs2435357** | **rs80227144** | **rs7005606** | **RSnc** | **RET nc risk^$^** | **ENS Gain** | **ENS loss** | **Zebrafish** | **Variant(s) found in indep. cohort** |
| --- | --- | --- | --- | --- | --- | --- | --- | --- | --- | --- | --- | --- | --- | --- |
| **P_000572** | TIA | Male | 1 | 0 | 1 | 0 | 0 | 1 | 1,00 | - | 0 | 0 | 0 | 0 |
| **P_000520** | Short | Male | 1 | 0 | 1 | 1 | 0 | 0 | 1,92 | ATT | 1 | 0 | 0 | 0 |
| **P_000540** | Short | Male | 1 | 1 | 1 | 1 | 0 | 1 | 2,98 | GTT or ATT | 0 | 3 | 0 | 0 |
| **P_000562** | Short | Male | 1 | 2 | 2 | 1 | 0 | 0 | 3,63 | GTT | 0 | 0 | 0 | 0 |
| **P_002459** | Short | Male | 1 | 1 | 2 | 0 | 1 | 1 | 3,77 | - | 0 | 1 | 0 | 0 |
| **P_000528** | Short | Male | 1 | 0 | 2 | 2 | 0 | 1 | 4,31 | ATT and ATT | 0 | 0 | 0 | 0 |
| **P_000537** | Short | Male | 1 | 2 | 2 | 1 | 0 | 2 | 4,57 | GTT | 0 | 12 | 1 | 3 |
| **P_000478** | Short | Male | 1 | 1 | 2 | 2 | 0 | 1 | 4,90 | ATT and GTT | 0 | 0 | 0 | 0 |
| **P_000561** | Short | Male | 1 | 1 | 2 | 2 | 0 | 1 | 4,90 | ATT and GTT | 0 | 19 | 1 | 1 |
| **P_001763** | Short | Female | 1 | 1 | 2 | 2 | 0 | 1 | 4,90 | ATT and GTT | 1 | 0 | 0 | 0 |
| **P_000482** | Short | Male | 1 | 2 | 2 | 2 | 0 | 0 | 5,01 | GTT and GTT | 0 | 0 | 0 | 0 |
| **P_000536** | abnormal | Female | 1 | 2 | 2 | 2 | 0 | 0 | 5,01 | GTT and GTT | 0 | 0 | 0 | 0 |
| **P_000555** | Short | Female | 1 | 2 | 2 | 2 | 0 | 0 | 5,01 | GTT and GTT | 0 | 0 | 0 | 1 |
| **P_000568** | Short | Male | 1 | 2 | 2 | 2 | 0 | 0 | 5,01 | GTT and GTT | 0 | 0 | 0 | 0 |
| **P_000573** | Short | Female | 1 | 2 | 2 | 2 | 0 | 0 | 5,01 | GTT and GTT | 0 | 1 | 0 | 0 |
| **P_002450** | Long | Male | 1 | 2 | 2 | 2 | 0 | 0 | 5,01 | GTT and GTT | 0 | 1 | 0 | 1 |
| **P_000553** | Short | Male | 1 | 2 | 2 | 2 | 0 | 1 | 5,48 | GTT and GTT | 0 | 0 | 0 | 0 |
| **P_002343** | Short | Male | 1 | 2 | 2 | 2 | 0 | 1 | 5,48 | GTT and GTT | 0 | 0 | 0 | 0 |
| **P_000567** | Short | Male | 1 | 1 | 2 | 1 | 1 | 2 | 5,63 | GTT or ATT | 0 | 4 | 1 | 3 |
| **P_002455** | Short | Male | 1 | 2 | 1 | 1 | 1 | 2 | 5,68 | GTT or ATT | 7 | 0 | 0 | 0 |
| **P_000494** | Short | Male | 1 | 2 | 2 | 2 | 0 | 2 | 5,95 | GTT and GTT | 1 | 0 | 0 | 0 |
| **P_000512** | Short | Female | 1 | 2 | 2 | 2 | 0 | 2 | 5,95 | GTT and GTT | 0 | 23 | 1 | 2 |
| **P_000559** | TCA | Male | 1 | 2 | 2 | 2 | 1 | 1 | 7,13 | GTT and GTT | 0 | 0 | 0 | 0 |
| **P_000302** | Short | Female | 2 | 0 | 1 | 0 | 0 | 0 | 0,53 | - | 1 | 0 | 0 | 1 |
| **P_002442** | Long | Male | 2 | 1 | 1 | 0 | 0 | 0 | 1,12 | - | 0 | 0 | 0 | 0 |
| **P_004502** | Short | Male | 2 | 0 | 1 | 1 | 0 | 1 | 2,39 | ATT | 0 | 0 | 0 | 0 |
| **P_000557** | TCA | Male | 2 | 0 | 2 | 1 | 0 | 1 | 2,92 | ATT | 1 | 0 | 0 | 0 |
| **P_000479** | Long | Male | 2 | 1 | 1 | 1 | 0 | 1 | 2,98 | GTT or ATT | 1 | 1 | 1 | 0 |
| **P_000518** | Short | Female | 2 | 1 | 1 | 1 | 0 | 2 | 3,45 | GTT or ATT | 0 | 0 | 0 | 0 |
| **P_000534** | Short | Female | 2 | 1 | 2 | 1 | 0 | 1 | 3,51 | GTT or ATT | 0 | 0 | 0 | 0 |
| **P_000570** | Short | Male | 2 | 1 | 2 | 1 | 0 | 1 | 3,51 | GTT or ATT | 0 | 0 | 0 | 0 |
| **P_000502** | Short | Female | 2 | 2 | 2 | 1 | 0 | 1 | 4,10 | GTT | 1 | 0 | 0 | 0 |
| **P_000526** | Short | Female | 2 | 2 | 2 | 1 | 0 | 1 | 4,10 | GTT | 0 | 0 | 0 | 0 |
| **P_000576** | Short | Female | 2 | 1 | 2 | 2 | 0 | 1 | 4,90 | ATT and GTT | 0 | 0 | 0 | 0 |
| **P_000566** | Short | Male | 2 | 1 | 2 | 2 | 0 | 2 | 5,37 | ATT and GTT | 0 | 0 | 0 | 0 |
| **P_000480** | Short | Male | 2 | 2 | 2 | 2 | 0 | 1 | 5,48 | GTT and GTT | 0 | 0 | 0 | 0 |
| **P_000486** | TCA | Female | 2 | 2 | 2 | 2 | 0 | 1 | 5,48 | GTT and GTT | 0 | 0 | 0 | 0 |
| **P_000544** | Long | Male | 2 | 1 | 2 | 1 | 1 | 2 | 5,63 | GTT or ATT | 0 | 0 | 0 | 0 |
| **P_000577** | Short | Male | 3 | 0 | 2 | 1 | 0 | 1 | 2,92 | ATT | 1 | 0 | 0 | 0 |
| **P_000515** | Short | Male | 3 | 1 | 2 | 1 | 0 | 2 | 3,98 | GTT or ATT | 0 | 0 | 0 | 0 |
| **P_001638** | Short | Male | 3 | 0 | 2 | 2 | 0 | 1 | 4,31 | ATT and ATT | 0 | 0 | 0 | 0 |
| **P_000578** | Long | Male | 3 | 1 | 2 | 2 | 0 | 1 | 4,90 | ATT and GTT | 0 | 0 | 0 | 0 |
| **P_000579** | Short | Male | 3 | 2 | 2 | 2 | 0 | 0 | 5,01 | GTT and GTT | 0 | 1 | 0 | 0 |
| **P_001635** | Short | Female | 3 | 2 | 2 | 2 | 0 | 0 | 5,01 | GTT and GTT | 0 | 0 | 0 | 0 |
| **P_000490** | Short | Female | 3 | 2 | 2 | 1 | 1 | 0 | 5,27 | GTT | 0 | 0 | 0 | 0 |
| **P_000498** | Long | Female | 3 | 1 | 2 | 2 | 0 | 2 | 5,37 | ATT and GTT | 1 | 0 | 0 | 0 |
| **P_000554** | Short | Male | 3 | 1 | 2 | 2 | 0 | 2 | 5,37 | ATT and GTT | 0 | 0 | 0 | 0 |
| **P_000505** | Short | Male | 3 | 2 | 2 | 2 | 0 | 1 | 5,48 | GTT and GTT | 0 | 0 | 0 | 0 |
| **P_000514** | Short | Male | 3 | 2 | 2 | 2 | 0 | 1 | 5,48 | GTT and GTT | 0 | 0 | 0 | 0 |
| **P_001639** | Short | Male | 3 | 2 | 2 | 2 | 0 | 1 | 5,48 | GTT and GTT | 0 | 0 | 0 | 0 |
| **P_000582** | Short | Male | 3 | 2 | 2 | 1 | 1 | 1 | 5,74 | GTT | 5 | 0 | 0 | 0 |
| **P_001636** | Short | Male | 3 | 2 | 2 | 2 | 0 | 2 | 5,95 | GTT and GTT | 0 | 0 | 0 | 0 |
| **P_000575** | Short | Male | 3 | 1 | 2 | 2 | 1 | 1 | 6,55 | ATT and GTT | 0 | 0 | 0 | 0 |
| **P_001632** | Short | Male | 3 | 1 | 2 | 2 | 1 | 1 | 6,55 | ATT and GTT | 1 | 0 | 0 | 0 |
| **P_000552** | Short | Male | 3 | 1 | 2 | 2 | 1 | 2 | 7,02 | ATT and GTT | 0 | 0 | 0 | 0 |
| **P_002431** | Short | Female | 3 | 1 | 2 | 2 | 1 | 2 | 7,02 | ATT and GTT | 0 | 2 | 0 | 0 |
| **P_000450** | Long | Male | 3 | 2 | 2 | 2 | 1 | 2 | 7,60 | GTT and GTT | 0 | 0 | 0 | 0 |
| **P_001637** | Short | Male | 3 | 1 | 2 | 2 | 2 | 1 | 8,19 | ATT and GTT | 1 | 0 | 0 | 0 |

*Depicted are the individual patient’s characteristics, number of predisposing haplotypes (as represented by the risk SNPs) and the noncoding risk score (RSnc). Additionally, the RET risk haplotypes as described in S7 are depicted. In blue the number of “ENS genes” in a CN gain, in red the number of “ENS genes” in a CN loss. Depicted in blue: (1) functional evidence from zebrafish studies and (2) the number of additional patients containing putative deleterious variants in genes within the rare CNVs. ^$^No phasing was performed to discern the most likely haplotype.*
