# Supplementary material for: Size matters: Large copy number losses in Hirschsprung disease patients reveal genes involved in enteric nervous system development
Source: PLoS Genet. 2021 Aug 6;17(8):e1009698. doi: 10.1371/journal.pgen.1009698 (PMC8372947; doi:10.1371/journal.pgen.1009698)
Supplement: S9 Table — (DOCX) [file pgen.1009698.s013.docx]

**S9 Table: Details of the gRNA sequences used**

| Gene | Position | Strand | Sequence | PAM | On-target score | Off-target score |
| --- | --- | --- | --- | --- | --- | --- |
| *tubb5* | 10322576 | + | GACCCAACCGGAACCTATCA | CGG | 69 | 96 |
| *gnl1* | 21665680 | + | TCGGCTGCTGTGACCTGACC | CGG | 62 | 95 |
| *tbx2a* | 56513198 | - | TTTCAAGGGTCTCGAGCCAG | AGG | 77 | 95 |
| *tbx2b* | 27365003 | - | GGTGGTGACTCAAAGCCGGA | TGG | 65 | 94 |
| *ufd1l* | 4768775 | - | GACCGCCCTCTTCCAACAAC | AGG | 56 | 96 |
| *usp32* | custom | - | CAGACGTTTGAGCTCCACAT | CGG | 70 | 82 |
| *akt3a* | 11233467 | - | CGGATACAAGGAGAAGCCAC | AGG | 96 | 97 |
| *akt3b* | custom | + | GTGAGTACATTAAGAACTGG | AGG | 83 | 83 |
| *gabbr1a* | custom | + | CTGGTTTAAGATCAAAGATC | CGG | 82 | 75 |
| *gabbr1b* | custom | + | GAACACAAGTTCATTCGAGG | GGG | 82 | 89 |
| *slc8a1a* | 30601171 | + | TCCCCAAAGGACGGGTTCAC | CGG | 77 | 97 |
| *slc8a1b* | 22381989 | - | CCAAGCGAAGACCACGCAAA | TGG | 72 | 95 |
| *mapk8a* | 31160246 | + | TAGTAACGGGTCACCACATA | TGG | 85 | 94 |
| *mapk8b* | 2516188 | + | CCACATTTCGATCGAGGACG | TGG | 61 | 98 |
