# Supplementary material for: Size matters: Large copy number losses in Hirschsprung disease patients reveal genes involved in enteric nervous system development
Source: PLoS Genet. 2021 Aug 6;17(8):e1009698. doi: 10.1371/journal.pgen.1009698 (PMC8372947; doi:10.1371/journal.pgen.1009698)
Supplement: S10 Table — (DOCX) [file pgen.1009698.s014.docx]

**S10 Table: Primers used for Sanger sequencing**

| Primer name | Primer sequence |
| --- | --- |
| tubb5_F | CCATAAAGCCTCCTGCTGAG |
| tubb5_R | TGGTAACAAATCTTTTCAACAATCA |
| gnl1_F | GCTGGTTGACGAGAGCTTG |
| gnl1_R | TGTTGGGGTCATATCTGCCG |
| tbx2a_F | AACCTTCGTTCTTTCCAGCG |
| tbx2a_R | AGAGGCTTCGATGCTATGTCA |
| tbx2b_F | CCCATGTCAGCTTTTCTCGC |
| tbx2b_R | TTCAATCGCGTAAACACTGC |
| ufd1l_F | TTCAGTGGAAAAGCGTGGTG |
| ufd1l_R | TGTGTCTTCAACTCTATCTGTGT |
| usp32_F | ACTTGAAGAATATCGCACGACT |
| usp32_R | GATGATGATCACGTTGAACTCAC |
| akt3a_F | TGTGCGATTGTGGGTTTGAG |
| akt3a_R | CCAGTAAAAGCAAGTCTCCAGT |
| akt3b_F | TGAACGTCGTGAAAGAGGGA |
| akt3b_R | GGAGTCTGATAATGAGAGCGAC |
| gabbr1a_F | CCGAGGCTTAACCGAGATT |
| gabbr1a_R | TCATGATCCTGTTGTGAAAGTCT |
| gabbr1b_F | CCTTTGGCGTATGATGCAGT |
| gabbr1b_R | GTCCTCGCCTGTGTGAACA |
| slc8a1a_F | TCCTATGAGCTCACGCCAGT |
| slc8a1a_R | TCTCCTGCGTCAAAGTTGTG |
| Slc8a1b_F | AACATCGCAAAGTGAAACACC |
| Slc8a1b_R | CTCCAGTTCCAAAGCCAGAG |
| mapk8a_R | ATCAGAGGGAGCACAAATGG |
| mapk8a_F | CGATTCTTTAGGACCTGAAACC |
| mapk8b_R | TCCATGTCTTACATTTTTGTGGTT |
| mapk8b_F | AATAAAGTGGCCGGTGAGTG |
