# Supplementary material for: Size matters: Large copy number losses in Hirschsprung disease patients reveal genes involved in enteric nervous system development
Source: PLoS Genet. 2021 Aug 6;17(8):e1009698. doi: 10.1371/journal.pgen.1009698 (PMC8372947; doi:10.1371/journal.pgen.1009698)
Supplement: S11 Table — (DOCX) [file pgen.1009698.s015.docx]

**S11 Table: Statistics, group sizes and gRNA efficiencies of zebrafish experiments**

|  | **HSCR scoring phenotype** | | | **# of neurons** | |  |  | **CRISPR/Cas9** | | **b variant of gene** | |
| --- | --- | --- | --- | --- | --- | --- | --- | --- | --- | --- | --- |
| **Gene name** | **significance** | **chi-squared** | **Power** | **significance** | **Power** | **#** | **Figure** | **Efficiency** | **R2** | **Efficiency** | **R2** |
| *slc8a1* | ***p=0,0073*** | 7,207 | 67.9% | ***p=0,0013*** | **96%** | n=15 | 3c-d | 84% | 0,90 | 10% | 0,95 |
| *mapk8* | p=0,0541 | 3,71 |  | ***p=0,0254*** | **95%** | n=34 | 3c-d | 85% | 0,89 | 17% | 0,84 |
| *tbx2* | P=0,0593 | 3,556 | 67.9% | p=0,0585 | 67.9% | n=25 | 3c-d | 77% | 0,86 | 57% | 0,89 |
| *gnl1* | p=0,4028 | 0,7 |  | p=0,0585 |  | n=30 | 3c-d | 75% | 0,80 |  |  |
| *ufd1l* | ***p=0,0166*** | 5,743 | 64.8% | ***p=0,0235*** | **90.5%** | n=27 | 3c-d | 77% | 0,91 |  |  |
| *usp32* | NA | NA |  | p=0,4586 |  | n=23 | 3c-d | 85% | 0,87 |  |  |
| *tubb5* | NA | NA |  | p=0,1869 |  | n=41 | 3c-d | 88% | 0,94 |  |  |
| *akt3* | NA | NA |  | p=0,937 |  | n=28 | 3c-d | 41% | 0,88 | 67% | 0,77 |
| *gabbr1* | p=0,3778 | 0,778 |  | p=0,6603 |  | n=27 | 3c-d | 80% | 0,89 | 82% | 0,82 |
|  |  |  |  |  |  |  |  |  |  |  |  |
| *mapk8* | ***p=0,0107*** | 6,518 | **78.4%** | NA |  | n=20 | 3e | 87% | 0,89 | 29% | 0,88 |
| *tbx2* | p=0,4073 | 0,687 |  | NA |  | n=38 | 3e | 92% | 0,92 | 27% | 0,97 |
| *gnl1* | p=0,0664 | 3,371 |  | NA |  | n=32 | 3e | 96% | 0,96 |  |  |
| *ufd1l* | p=0,3915 | 0,734 |  | NA |  | n=31 | 3e | 96% | 0,96 |  |  |
| *usp32* | p=0,1847 | 1,759 |  | NA |  | n=32 | 3e | 85% | 0,91 |  |  |
| *tubb5* | ***p=0,0003*** | 13,291 | **96,1%** | NA |  | n=58 | 3e | 86% | 0,86 |  |  |
| *akt3* | P=0,4157 | 0,663 |  | NA |  | n=30 | 3e | 51% | 0,92 | 54% | 0,82 |
| *gabbr1* | p=0,1481 | 2,091 |  | NA |  | n=31 | 3e | 81% | 0,81 | 89% | 0,90 |
|  |  |  |  |  |  |  |  |  |  |  |  |
| *tbx2* | ***p=0,0373*** | 4,339 | 54.2% | p=0,7402 |  | n=33 | 4a | 95% | 0,95 |  |  |
| *ufd1l* | ***p=0,0208*** | 5,344 | 66.9% | ***p<0,0001*** | **100%** | n=40 | 4b | 86% | 0,86 | 68% | 0,91 |
| *mapk8* | ***p=0,0022*** | 9,346 | **94.1%** | p=0,1356 |  | n=37 | 4c | 87% | 0,89 | 29% | 0,88 |
|  |  |  |  |  |  |  |  |  |  |  |  |
| *gnl1* | ***p=0.0405*** | 4 | 55.3% | NA |  | n=23 | 5a | 96% | 0,96 |  |  |
