# Supplementary material for: Size matters: Large copy number losses in Hirschsprung disease patients reveal genes involved in enteric nervous system development
Source: PLoS Genet. 2021 Aug 6;17(8):e1009698. doi: 10.1371/journal.pgen.1009698 (PMC8372947; doi:10.1371/journal.pgen.1009698)
Supplement: Attachment — Submitted filename: Reviewers_comments_CNV.docx [file pgen.1009698.s017.docx]

**Dear Editor,**

**We are pleased to re-submit our manuscript” Size matters: large copy number losses in Hirschsprung disease patients reveal genes involved in enteric nervous system development.” for publication in PLOS Genetics. We thank the editor and reviewers for their constructive comments, which have helped us to improve the manuscript. We have extensively revised the manuscript and responded to the reviewers comments in the attachment (response to reviewers), revisions are highlighted in the manuscript (green).**

**We sincerely hope you will accept our revised manuscript for publication in your journal.**

**Kind regards,**

**Laura Kuil & Erwin Brosens**

**Response to reviewers**

**:**

Major comments

1. The information needed to understand the experimental design and results is not where it needs to be and instead is buried within the supplemental material or in the methods, including some of the main findings. The main figures and supplementary figures lack appropriate legends. And the supplementary material is not well organized and much of it is not explicitly referred to in the paper. Some examples:

***We agree and we revised the manuscript extensively and we moved a lot of information from the supplement to the main manuscript to improve readability.***

a) The rare CNV analysis is not properly introduced. Key to this analysis is the classification of subgroups of HSCR-AAM. Figure 1A/B/3, depict HSCR patients classification into 3 groups, however the results and figure legends never describe these groups. This information is found only in the “Patient Inclusion” section of the methods on page 8, and needing to search for this important information affects readability.

***We agree with the reviewer and we now included the information about the groups of patients in the first section of the results.***

b) The evidence for the first finding, “The number of rare CNVs per individual did not differ between subgroups or controls (S4).”, appears to not be represented in the figure, but only in the S4 legend text. This is one of the key findings cited in the abstract, and should be included in the main text and/or be clearly represented by a figure data panel (i.e. S4n), instead of needing to hunt for the information. Likewise, data for another main finding cited on p4 “In Group 1, rare CNVs are enriched for ENS genes (p=4.565E-6, S7).” appears not to be within S7, but in S4, but again only in the figure legend. And it isn’t clear what data this point refers to. Again, these main findings should be clearly presented.

***The number of rare CNVs is now included in main figure 2A (not significant). In addition, we now present all relevant plots in the main figure and made major revisions in the text to improve this explanation and refer for S4 for the statistics.***

c) Further CNV analysis is found over the 1 table and 10 plots of S4. Each piece of data should have designators (i.e. S4n) and the finding from each plot indicated in the results section. Many of these results are currently only discussed within this supplementary figure legend. If these results are not presented in the paper, then they should not be included. The same is true for all data figures and supplemental data figures. Careful consideration must be made of what to include and to properly describe any included data.

***Thank you for your comment. We now included the relevant graphs in main figure 2. In addition, we mention all the graphs in the text in the correct order. Furthermore, we made major adjustments throughout the text and implemented most of the text from the supplementary files into the main text to improve readability.***

d) Acronyms such as RS (and RSnc, RScnv and RSrcv) are not defined in the legends. Nor do they appear in the text on first appearance (i.e. not for Table 1, S4). RSnc eventually described in reference to Fig2C and S11 (see also point 1, above). The other acronyms are never described or referred to other than appearing in Fig1b. In Tables 1 and 2, the acronyms in the column headers are not defined. A few are also mentioned in S9, but still not defined.

***We now defined all the acronyms upon first appearance.***

e) The term “ENS genes” is used in the results on page 4 in reference to S7 (and in S4) but without explanation. After searching, a partial definition of this term is found within the legend of S4 and also obliquely described in the methods page 9. This is a key piece of information that should be clearly described in the relevant part of the results section.

***Thank you for pointing this out, we now describe this clearly in the results and material and methods section as seen below***

***Results section:***

***A total of 230 genes affected by a rare CNV, had higher mRNA expression in the developing mouse ENS compared to the whole intestine (n=91 in group 1-3, n=139 in group 4) [39, 40]. We considered these genes to be important for ENS development and will refer to those genes as “ENS genes” throughout the paper.***

***Material and methods section:***

***and higher expressed in the developing mouse ENS compared to whole intestine, between embryonic day E11.5 and E15.5 (referred to as: ENS genes)***

2. Several issues relate to the ENS phenotypes described following CRISPR/Cas9 targeting of zebrafish genes:

a) Phenotypes are described variously as “hypoganglionosis”, “ultra-short segment HSCR”, “short segment HSCR” and “total colonic HSCR”. This is not a valid description of these zebrafish phenotypes. There is no justification for the use of these terms and they are highly misleading. The whole zebrafish intestine is not equivalent to the mammalian colon. Instead, phenotypes affecting the distribution of ENS neurons along the gut length should be presented in neutral terms/values that actually describe the phenotypes. While the term “hypoganglionosis” has been used previously to describe zebrafish ENS phenotypes with a reduction in ENS neuron number, this term is not accurate. The zebrafish ENS has no ganglia. A proper descriptive term should be used.

***We agree that indeed it is not justified to simply state that the zebrafish show aganglionosis, since indeed zebrafish neurons do not form ganglia. Therefore we now also mention this in the text and we included a clear description of our classification strategy that is now also published here: Kuil LE , Chauhan RK, Cheng WW, Hofstra RMW and Alves MM. Zebrafish: a model organism for studying enteric nervous system development and disease. Front Cell Dev Biol 2020. 10.3389/fcell.2020.629073***

b) It is not stated how a designation of “hypoganglionosis” is given. Are neurons counted? What percentage reduction constitutes a phenotype?

***See comment above. In addition, we now counted the neurons and normalized the counts to the length of the intestine as an additional readout next to the classification.***

c) Are phenotypes observed outside of the ENS? Are any growth abnormalities observed in any gene targeting experiment? This is an important point since general developmental delays would affect the number and distribution of neurons observed at 5dpf.

***We now mention phenotypical abnormalities in the text and added two supplemental figures with brightfield images of the larvae showing the presence or absence of other morphological aberrations. Morphological abnormalities was only a concern in the case of slc8a1, since the majority of fish showed malformations and severe pericardial edema.***

d) P-values are given for observed phenotypes in S16. It is unclear whether all types of phenotypes have been lumped together for these statistics.

***Yes we used the percentage of fish presenting with a “normal” formed ENS for the statistical analysis. We now state this in the text as well.***

e) Results and p-values are described for "tbx2" in Figure 2D and S16, but it is unclear if this refers to CRISPR/Cas9 targeting of tbx2a or tbx2b or a combination. The same question arises for gabbr1a/1b, slc8a1a/1b and mapk8a/8b.

***We now clearly state in the text that in all cases both zebrafish variants (a and b) orthologue to the human candidate genes were disrupted simultaneously.***

f) Ns are low for all CRISPR/Cas9 experiments, ranging from 15 to 40 embryos per gene/gene pair targeted. In the case of slc8a1, a p-value of 0.0073 is given for an experiment involving 15 embryos. Power calculations should be shown to demonstrate the appropriate number of animals needed for statistically relevant results, and that the numbers used in these experiments are sufficient.

***We based our experiments on power calculations that showed that with an alpha of 0,05 an incidence of “HSCR” phenotypes in 75% of the cases the groups group sizes of 26 would be sufficient for a power of 80%. However, in the case of slc8a1 many of the larvae showed such severe malformations that we excluded them from analysis. This resulted in a high dropout rate. Post-hoc power analysis did show that upon quantification of individual neurons the low number of larvae were sufficient to induce a significant effect with a power of 96%. The post-hoc calculated power for all statistically significant findings (p<0.05) is now included in S15 together with the p values.***

g) The description of the results in Figures 2E and 2F is very sparse and requires further elaboration and discussion.

***We agree and made major revisions throughout the manuscript and elaborated more on the zebrafish experiments and results.***

h) The phenotypes observed in zebrafish should never be described as “HSCR” (i.e p7 of the Conclusions). See point 1a, above.

***See response above (2a)***

i) Candidate HSCR disease genes should be described as “candidates” rather than “HSCR disease genes” (see p. 8, for example)

***We adjusted this throughout the text.***

j) A p-value is given for ret+tbx2, but no results are shown in Fig2e and no data is shown for slc8a1

***Thank you for pointing this out, tbx2 data was not included by accident we now included the correct graph. We also now mention in the text that we did not include slc8a1 in the ret morpholino experiments to the severe malformations we observed already upon disruption of the genes alone, which led to a high dropout.***


3. Other issues:

a) Figure 1 is a graphical display of the experimental design. The images chosen are mostly far too small to see. Key information is missing (i.e. what are the subgroups, see point 1a, above), and some images are possibly not appropriate (‘ENS’ panel suggests sequencing gels were run? Wasn't this panel referring to microarray data?). This entire figure requires significant improvements to be useful.

***Figure 1 is a schematic representation of our study set-up. We adjusted figure 1, now we included the subgroups of HSCR patients in panel A. The “ENS” panel represents a heatmap of differential expression, the microarray data of differential expression between ENS and remainder of the mouse intestine was one of the criteria to be considered an “ENS gene”. We revised the figure and included more detailed descriptions in the figure legend.***

b) Why isn’t ChAT chosen for analysis (Table 1)? From the logic presented, it’s not clear why this gene is not selected, for it features in S7 and is an ENS expressed gene.

***Indeed CHAT is enriched in the ENS and is important for proper ENS functioning. We cannot rule out an effect on ChAT expression, however this gene is not under constraint and therefore not selected as a candidate gene. Variant intolerance is checked in gnomAD for example:*** [***https://gnomad.broadinstitute.org/gene/ENSG00000070748?dataset=gnomad_r2_1***](https://gnomad.broadinstitute.org/gene/ENSG00000070748?dataset=gnomad_r2_1)

c) The statistics designations (i.e. meaning of ** vs ***) are never explained in the methods. The actual statistics should be found in the figure or in the figure legend, in addition to the main text. This is especially important because some results are not described in the text (i.e. most of S4 plots where * designations are not given, though p-values are indicated in the legend. But it’s unclear which p-value is referring to which panel - see point 1c, above).

***We now included in the methods and in the figure legends the meaning of the designations (* p < 0.05 ** p < 0.01 *** p < 0.001). In the main text the actual p-values are given and in the figure legends and materials section we refer to the proper supplemental file containing the statistical information.***

d) The term “overexpressed” is used many times, for example in “a constraint gene overexpressed in the mouse ENS” (p.9) , but these genes are NOT overexpressed in the ENS. This term suggests some alteration or manipulation. Instead, these genes are expressed in the ENS. They are also expressed in the ENS more abundantly than other reference tissues (i.e. other gut tissues). This term needs to be changed throughout the text/figures/legends/data tables, etc..

***Thank you for your comment, we understand now that the description is confusing. Indeed we mean that the gene expression is higher in the ENS compared to total intestine but is not artificially overexpressed of course. We adjusted this by introducing what we consider to be an ENS gene in the results and material and methods sections as can be seen below.***

***Results section:***

***A total of 230 genes affected by a rare CNV, had higher mRNA expression in the developing mouse ENS compared to the whole intestine (n=91 in group 1-3, n=139 in group 4) [39, 40]. We considered these genes to be important for ENS development and will refer to those genes as “ENS genes” throughout the paper.***

***Material and methods section:***

***and higher expressed in the developing mouse ENS compared to whole intestine, between embryonic day E11.5 and E15.5 (referred to as: ENS genes)***

e) On p5 the statement: “However, there was no significant enrichment for nonsense, splice site or missense variants in genes impacted by rare gains nor losses (S9)" seems at odds with the data in S9, which shows p-values of <0.05 for 5 genes.

***Indeed, the first five genes have a p-value under 0.05. We thank the reviewer for pointing this out. In a rare variant burden test, a p-value 0f 0.05 is not yet considered to be significant, as a multiple testing correction has to be applied. Given the sample size and possible variant positions tested we ranked the genes based on p-value and considered a p-value below 1x10e-5 of interest. We did not mention this in the text and added this. Furthermore, four out of five genes are more affected in controls compared to patients. TNRC18 is a gene under constraint, it has a high PLI (but a negative z-score for rare missense variants). It does not fit the criteria for “ENS gene” and is affected by a CN Gain (not a loss). We did not detect LOF variants in this gene.***

***We added this sentence to S9: “Given the sample size and possible variant positions tested we ranked the genes based on p-value and considered a p-value below 1x10e-5 of interest”***

f) S15 appears to refer to figures incorrectly.

***Thank you for pointing this out, we now corrected the figure references.***

g) Attention to writing is needed throughout, and especially in the author summary.

***We agree and made major revisions throughout the manuscript.***

Reviewer #2: 

This article would immensely benefit form a comprehensive revision and organization of the material presented. Paper is not well organized and critical information is buried in the supplemental files without proper descriptions and annotation links from within the main paper. The paper would benefit from editing for verb tense and typographical errors. The manuscript as currently presented detracts from the data presentation.

***We agree and we revised the manuscript extensively and we moved a lot of information from the supplement to the main manuscript to improve readability.***

From the start of the results section, the authors should clearly define the patient grouping characteristics upfront. This identification needs to be clearly stated in the main text and in the appropriate figure legends, not merely placed at the end, in the methods, patient inclusion section.

***Thank you for the suggestion, we now start the results section with a description of the groups and also included this is Figure 1 panel A.***

With respect to the assessment of gene function in zebrafish, full images representing gene knockdown embryos should be included in supplement as this data would be of interest to a broader audience. I would note that under the supplement section for patient P_000479, the authors do state “In line with this, we show that zebrafish larvae where slc8a1a and slc8a1b were disrupted, severe heart edema was present, as well as swollen heads”, however images of this data was not presented.

***Thank you for the suggestion, we now included two supplemental figures containing several brightfield images of the slc8a1 phenotypes we observed and also representative images from larvae injected with all the other gRNAs.***

Authors should clearly note which of the two alleles for each gene are being displayed in the Figure 2f gRNAs plots. For each of these genes the authors may find helpful the ZFIN resource (zfin.org). This resource will allow for identification of published results of previously characterized Zebrafish gene expression and allele specific phenotypic results for comparison. These phenotypes should be included when discussing the gene expression or phenotypes alongside those of mouse models as the authors are utilizing zebrafish as their research model for phenotypic evaluation.

***We now clearly state in the text that in all cases both zebrafish variants (a and b) orthologue to the human candidate genes were disrupted simultaneously. We also placed additional references to published work on the zebrafish mutants with respect to their non-ENS phenotypes in the results and discussion sections.***

In supplemental section 8, authors utilize this section to not only present patient phenotypic information, but also expand on data presented and draw conclusions based on this data. Much of the information included here would benefit from a rewrite consolidation and reworking into the discussion of the main paper.

***Thank you for the suggestion, we agree with this and moved the discussion of the candidate genes and findings in animals models to the discussion and extended this with zebrafish data as well (see comment above).***

Authors should address in their discussion the large number of individuals remaining without an explained genetic diagnosis following this analysis including the extent to which complex rearrangements will remain undetected by SNP array-based analysis (eg. complex rearrangements without copy number alterations such as inversions and the need for better resolution of breakpoint junctions with respect to genes in the cases of duplications). The number of patients without definitive diagnosis is not unique to HSCR patients and limitations of the approach taken should at least be acknowledged. While authors have focused on rare CVNs with associated with region loss, roughly 50% of the rare CNV’s detected in table S3 represent duplications. Are these duplications included in the data for figure 3b and if so, do the authors have evidence of gene disruption at breakpoints or that upregulation of critical genes contained within the intervals contribute to HSCR?

***We thank the reviewer for highlighting this. Indeed, balanced rearrangements are not detected with SNP-array analysis and the resolution to detect copy number changes varies of the genome and has its limited. Therefore, we focused on rare CNV with stringent quality settings addressing probe number, threshold settings for logR ratio and B-allele frequency etc. (see ref EJHG, Brosens et al 2016) and we visually inspected these profiles to exclude likely technical artefacts.***

***During our statistical analysis, CN losses – not CN gains – proved to be enriched in HSCR-AAM. This does not exclude the contribution of CN gains, we merely did not find statistical evidence for their contribution in our cohort. Therefore, we did not focus on the genes within these gains in zebrafish studies, and did not include them in the data for figure 3b. Indeed, genes could be disrupted (either directly or via inversion-duplications) within the gains observed (and as a consequence be loss of function structural variants) or regulatory elements could be misplaced. We did search for disrupted TAD borders or genes within our data. However, the resolution of our SNP-array data was not sufficient to detect this confidently, nor did we have the resources to perform additional experiments to investigate this. As we strongly agree with the reviewer that gains can indeed be of consequence and explain a proportion of the HSCR phenotypes seen in this cohort we added the following sentences:***

“We were able to pinpoint and associate several genes within a Copy Number loss to the HSCR phenotype seen in these patients. However, this is most likely an underestimation of the true contribution of CNV to HSCR, since SNP-array has relative limited resolution to detect small CNV and is not able to detect balanced structural variations as inversions and inversion-duplications. Approximately 50% of the rare CNVs we find are gains. As only CN losses – not CN gains – proved to be significantly enriched in this HSCR-AAM cohort, we did not include CN gains in our screen. In future studies, we would recommend the use of whole genome sequencing (WGS) approaches, as these are able to confidently detect much more structural variant types and provide more patients with a definite genetic diagnosis. The benefits of WGS approaches would include the detection of inversion-deletions (and disrupted genes within a gain), more precise breakpoint mapping, the detection of balanced rearrangements (and disruption of TAD borders and/or regulatory elements by CN losses or Gains), or a second mutation in a gene impacted by a CNV on the other allele (unmasking a recessive mutation or variant). Screening at improved resolution and ability to detect a wider range of genetic aberrations would likely increase the number of associated genes in the remaining patients without an explained genetic diagnosis.”

Minor points.

Authors use the term “constrain” and “constraint” inappropriately throughout the text. These instances should either use the terms “genes under constraint” or “constrained genes”.

***We corrected this throughout the paper.***

All figures should be numbered to reflect the order that they first appear in the text. For example, supplements S1 and S2 are not mentioned in the main text.

Figure 2. All asterisks in the figures should be appropriated defined in the respective figure legends.

***We substantially improved the figure legends and included the definition of the asterisks.***

Figure (2a). Label y- axis clearly, is it bp or Mb?
***Labelled the axis correctly (bp)***

Figure (2d and e). The total number of animals used should be included in this figure/figure legend. At a minimum, authors should indicate within the figure 2 legend that the total number of animals evaluated is presented in S16. In addition, authors should clarify if the P-value presented in figure 2e is reflective of the asterisk to the right or does it indicate a P-value for the bars below.

***We extended the figure legends and refer to supplemental table where we listed the total numbers of fish per group.***

Figure (2f). In the top panel, the fish are labeled “normal”, however this is then presented with a number 15/23. I assume the authors wish to convey that these are the “control” individuals with a normal distribution pattern. This should be better described in the figure legend.

***We made major adjustments to the figure legends to make this clear. These are injected fish that present with a “normal” ENS.***

The term CN should be defined in the text at first usage.

***Thank you, we now defined CN at first usage.***

S4) A subset of graphs presented here appear to be the same as those already included in Figures 2a, 2b and 2c.

***We made major adjustments to the figures and removed the duplicated graphs***

S6) Please define the headers for this table.

***We now defined the headers in the table legend.***

S16) Consistent capitalization/non-capitalization should be utilized (“p”-value and “chi” in S16 , and supplement index).

***Thank you for pointing this out. We corrected this now.***
