# Supplementary material for: Size matters: Large copy number losses in Hirschsprung disease patients reveal genes involved in enteric nervous system development
Source: PLoS Genet. 2021 Aug 6;17(8):e1009698. doi: 10.1371/journal.pgen.1009698 (PMC8372947; doi:10.1371/journal.pgen.1009698)
Supplement: Attachment — Submitted filename: LK_EBR_REBUTTAL_22052021.docx [file pgen.1009698.s018.docx]

**REVIEWER 1:**

1) The terms “hypoganglionosis”, “ultra-short segment HSCR”, “short segment HSCR” and “total colonic HSCR” do not belong in the Results or the Figures. The fact that these authors have published their proposed classification strategy in a Review article (Kuil 2020) is immaterial to the point made here. This classification is not accurate or justified (the whole zebrafish intestine is not equivalent to the mammalian colon), is misleading and does not belong in a research paper Results section. Neutral, descriptive terms should be used in the Results to describe the findings. If the authors wish to raise in the Discussion the possibility that zebrafish phenotype severity may relate to HSCR phenotype classifications, and allude to their review article, this would be acceptable. The use of these terms is not only provocative and inaccurate, but also entirely unnecessary for this study, since the effect of gene disruptions are only described as having “significant effect” or “effect” or “significant increase in percentage of larvae that showed a phenotype”. The use of inappropriate terms offers no additional information and only clouds a simple analysis. Further to this general point, the terms “HSCR penetrance in zebrafish” and “HSCR penetrance” should not be used in the Results. The Discussion is the appropriate place to raise these speculative comparisons.

***We now categorized the different phenotypical presentations by 5 categories. We made use of neutral descriptions of the phenotypes now in the figures, methods and results.***

2) The term “overexpressed” is not accurate to describe genes more abundantly expressed in the ENS relative to a reference tissue, and should not be used. These genes are “expressed” in the ENS. The changes made to the manuscript do not address the issue raised.

***We agree with reviewer 1, it is better to use a different description of the elevated expression. We used the term “overexpressed” to describe the higher expression in the isolated ENS cells compared to the intestine. Micro-array measures relative expression and as a consequence we can only say that a gene is higher or lower expressed compared to a reference (the intestine). We changed the terminology to “expressed” , “higher expressed” or “upregulated” where applicable.*** ***Also, see our answers to the next 2 points.***

3) In Figure 1, the schematic representation of the study set-up, the image used in Fig 1B to represent microarrays appears to be of a sequencing gel, though the legend states that this is intended to represent a heat map. This image is unrecognizable as a heat map. It is likely that the authors did not amend the figure to include the BRB-ArrayTools image, as was indicated in their rebuttal letter. Importantly, were heat maps used to identify differentially expressed genes? If so, this analysis should be shown. The methods suggest that straightforward differential expression analysis was used, presumably with some (unspecified) fold-change cut-offs, in which case heat maps should be shown to depict those genes that met this fold change cut-off, with an accompanying data table. Or perhaps scatterplots with fold change cut-offs shown and an accompanying data table. In the schematic, another more appropriate image should be found to represent microarray analysis, and best would be to select an image that reflects the analysis that was actually done. And any relevant analysis should be shown as a figure and/or data table. And the methods should be properly described in the Methods section. See also points 5 and 6 below.

**In the overview method figure we aimed to present a schema or overview of the method used. The heatmap we used (it’s not a sequencing gel) is supposed to be a description /symbolic flowchart of the methods used (as sequencing reads could be for sequencing technologies)**

**Below the brb-array figure for known disease gene analysis as an example. Brb-array tools uses the colour shades of blue as a standard setting for their heatmaps. We now used this figure in the graphical overview. We incorporated this figure (with known HSCR disease genes) as an example of gene expression analysis in the workflow.**

**We are not sure how a heat map can be used to identify differentially expressed genes, we use it as a visual aid to distinguish (sub)groups. Instead of referring to the corresponding manuscript, we now describe the method we used to process the raw intensity files and to analyze the genes affected by a rare CNV (by evaluating a “CNV gene panel”) in brb array tools. We added this text to the method section:**

**Evaluation of candidate gene expression**

**We prioritized candidate genes based on gene characteristics: genes that are intolerant to variation and/or dosage sensitive (CCR; see Variant prioritization in the next paragraph), and higher expressed in the developing mouse ENS compared to whole intestine, between embryonic day E11.5 and E15.5, referred to as “ENS gene” [41, 42]. Genes affected by a rare CNV were extracted from the CNV regions and converted to mouse orthologues to make a gene panel for downstream analysis (S15). Gene expression data used to determine the “ENS genes” was downloaded from the gene expression omnibus (GSE34208 and GSE100130). We calculated probe set summaries from the raw Affymetrix data using BRB-ArrayTools version 4.5.0 - Beta_2 (http://linus.nci.nih.gov/BRB-ArrayTools.html) and used the “Just CCRMA” algorithm to adjusts for background intensities. We normalized each array using quantile normalization that includes variance stabilization and log2 transformation. Replicate spots within an array were averaged. Genes showing minimal variation across the set of arrays were excluded from the analysis (if less than 20% of expression data had at least a 1.5 fold change in either direction from a gene's median value, or at least 50% of arrays had missing data for that gene). The minimum fold change for the class comparisons was set at 1.5 and differential expression was determined using a two-sample T-test with random variance model. The permutation p-values for significant genes were computed based on 1,000 random permutations and the nominal significance level of each univariate test was set at 0.05. Subsequently, we defined “ENS genes” as genes higher expressed in isolated “ENS cells” (with and without the addition of GDNF) compared to the expression in the total intestines. These genes were considered as “ENS genes” and included in downstream analysis. As only micro-array-based expression data of the mouse ENS was available, we cannot reliably distinguish between not expressed and not differentially expressed genes between the ENS and the intestine. Unfortunately, there was no data available from human intestines during ENCC migration. However, RNA sequencing data from human colon was available for embryonic week 12, 14 and 16 (GSE111307)[95], we annotated the gene expression during these time points for reference to S15. We used the settings and methods described previously, as well as the previously generated and publicly available datafile “GSE111307_ENS_development_lenient_mapping.xlsx” at https://www.ncbi.nlm.nih.gov/geo/query/acc.cgi?acc=GSE111307.**

4) The term “ENS gene” is still used before being defined (p. 8, lines: 170, 172, 177, 181), meaning that the logic and readability is still an issue in this version. Is this term even intended to be the same as “ENS gene” as defined on p9? Or is there some other criteria for the term used on p8? If different, this term should also be explained. If the terms are the same, then the definition must come earlier in the text. Moreover, the term as currently defined on p9 is not clear. Presumably an “ENS gene” is one which has higher expression in the developing mouse or human ENS compared with the whole intestine. As written, the definition refers to a sentence also describing CNVs, which is unnecessarily confusing: “Whereas a total of 230 genes affected by a rare CNV, had higher mRNA expression in the developing mouse ENS compared to the whole intestine (n=91 in group 1-3, n=139 in group 4) [43, 44]. We considered these genes to be important for ENS development and will refer to those genes as “ENS genes” throughout this manuscript.” This definition should be simply and clearly stated.

**Thank you for pointing this out. We apologize for not explaining at first instance.**

**In line 169-172 we now define the ENS gene term as:**

**“****We used a previously published microarray dataset and considered genes as “ENS genes” if the gene expression is higher in isolated “ENS cells” (with and without the addition of GDNF) compared to the expression in other intestinal cells or total intestines of mice [41, 42].”**

**And as well in the method section Line 531-533:**

**“****Subsequently, we defined “ENS genes” as genes higher expressed in isolated “ENS cells” (with and without the addition of GDNF) compared to the expression in the other intestinal cells or total intestines.”**

5) The section “Candidate genes with a loss are under constraint and expressed during ENS development” is very confusingly written. The first indication that expression of genes is being examined comes from: “Of these, 514 did not have a known mouse orthologue or probes in the microarray datasets used”. But nowhere is the use of microarray datasets explained in the Results. Certainly the schematic representation of the study set-up in Figure 1 does not adequately present or describe this part of the study (see also point 3, above) and nor does the cursory figure legend text: “The genes included in the CNVs were ranked based on their expression in the developing mouse ENS relative to that of the total intestine (for illustrative purposes a screenshot of a heatmap of differential gene expression derived from brb-array tools (<https://brb.nci.nih.gov/BRB-ArrayTools/> )”. Information to understand this study belongs in Results section (see point 3, above). There is no reason not to describe this work simply and clearly.

**We agree, we have rewritten this section substantially and included methods for the expression analysis as well as the figure legend.**

6) There is no data table corresponding to the “Evaluation of candidate gene expression” to show the complete list of genes this analysis identified as expressed in the mouse ENS or the human embryonic colon. See point 3, above. This is required to show that the analysis was performed correctly and, importantly, to show qualitative expression information for the ENS expressed genes followed up in this study.

**We have added the table S15 to the supplementary data. This excel sheet contains two worksheets, one with the human genes present in rare CNV (in patients and controls) annotated with mouse orthologues, human embryonic gene expression values, whether or not upregulated in mouse ENS or not and if a gene would qualify as a “constrained coding region” There is also a worksheet describing the zebrafish orthologues.** **In addition, we described our analysis in the material and methods section (see our answer to comment 3).**

7) There is no experimental basis for speculating a role for TUBB in HSCR, since no effect was seen in Crispr/CAS9 experiments targeting tubb1. Therefore, this cannot be stated as is written (p16): “Although we did not find an (e)affect on ENS development in zebrafish, the absence of enteric neurons in the distal colon and other neurological symptoms seen in Patient P_000512, can be likely related to the deletion of TUBB and subsequently to an increased neuronal apoptosis or perturbed neuronal migration or differentiation in combination with the effects of GNL1.” Importantly, in fact, the zebrafish orthologue of TUBB is tubb5, although tubb1 (the TUBB1 orthologue) was tested in these experiments. So it appears that the right experiment has not been done in any case. If so, this would suggest that no conclusion can be made on the role of TUBB in HSCR and the Results and Discussion should be modified accordingly. Finally, the Discussion (in general) is very long, and especially so in this section discussing the lack of evidence for roles of GABBR1 and TUBB.

**We agree and thank you for pointing this out once more. In previous annotations the human gene was called TUBB1 or TUBB5, hence the confusion around the conserved genes. With the current biomart (ENSEMBL) zebrafish tubb5 is indeed the correct orthologue of human TUBB. We now did additional experiments to test the zebrafish tubb5 gene, which revealed that tubb5 alone does not affect ENS development, but does show epistasis with ret. Therefore, our results and conclusions are now adjusted accordingly. Upon your suggestion we also shortened the discussion.**

8) A table should be provided to show the orthologous Mouse and Zebrafish genes for the 1216 Human genes or transcripts present in CNVs of group 1-3 and group 4, to include the supporting source reference for these orthologue assignments (i.e. HGNC, MGI, ZFIN, Ensembl). This is important for point 7, above. Moreover, together with the table indicated in point 6, this information is necessary to provide support for statements such as “Of these, 514 did not have a known mouse orthologue or probes in the microarray datasets used” (p.9, line 201). Given the fact that 99% of human genes have mouse orthologues, it is unlikely that ~half of the 1216 human genes or transcripts lack mouse orthologues, and more likely that the many of the orthologous mouse genes were not present on the arrays. The table should provide information to allow these two possibilities to be clearly distinguished. This should also be discussed in the “Limitations and discrepancies” section.

**We have added a supplementary table (S15) to the supplementary data files after** **having re-evaluated the dataset used using the latest version of ENSEMBL biomart (previously we used MGI). Most “genes” still do not have a mouse orthologue. This is however mostly due to the concept of “gene and transcripts ” we used. Almost all missing “genes and transcripts” were microRNA, long noncoding RNA or were genes of which the symbol is not available (e.g. AK308704, BC112340, DQ594736, HCG20, LOC105369423). We have made a distinction between these types of elements, genes not expressed and genes without a probe in the micro-array). Using the newer version of the ensemble reference genome and including synonyms we were able to add 14 mouse orthologues to the dataset. None of these genes were Constrained Coding Region (CCR) and often present in both patients and controls. These are marked in orange and as not evaluated in S15.**

9) The authors have addressed a comment from Reviewer 2 regarding “constraint”, but in doing so have now made further syntax/grammatical errors: i.e. “genes under constrained” throughout (i.e. Author Summary, Results, Discussion). In general the Author Summary continues to require attention to writing. Punctuation and other grammatical/syntax errors are an issue throughout the entire text and affects readability. Note also “Danio rerio”. The Discussion would benefit from shortening.

**We changed the term (genes under constraint) to “****Constrained Coding Region (CCR)” throughout the text. We also rewrote parts of the manuscript to improve the writing.**

**REVIEWER 2:**

The definition if the term “ENS genes” should be made at the first usage of the term online 170, rather than where it is later defined on line 207.

S1. Correct spelling of undetermined

***Thank you, we corrected this.***

S8. Column 1 header is missing the “t” in patient
Text in table description should include apostrophe to be patient’s

***Thank you, we corrected this.***

S9. Capitalize “Primer”

***Thank you, we corrected this.***
